# Supplementary material for: The Incidence of Myocarditis and Pericarditis in Post COVID-19 Unvaccinated Patients—A Large Population-Based Study
Source: J Clin Med. 2022 Apr 15;11(8):2219. doi: 10.3390/jcm11082219 (PMC9025013; doi:10.3390/jcm11082219)
Supplement: Supplementary file 1 [file jcm-11-02219-s001.zip › jcm-1674243-supplementary.pdf]

Supplementary Figures and Tables

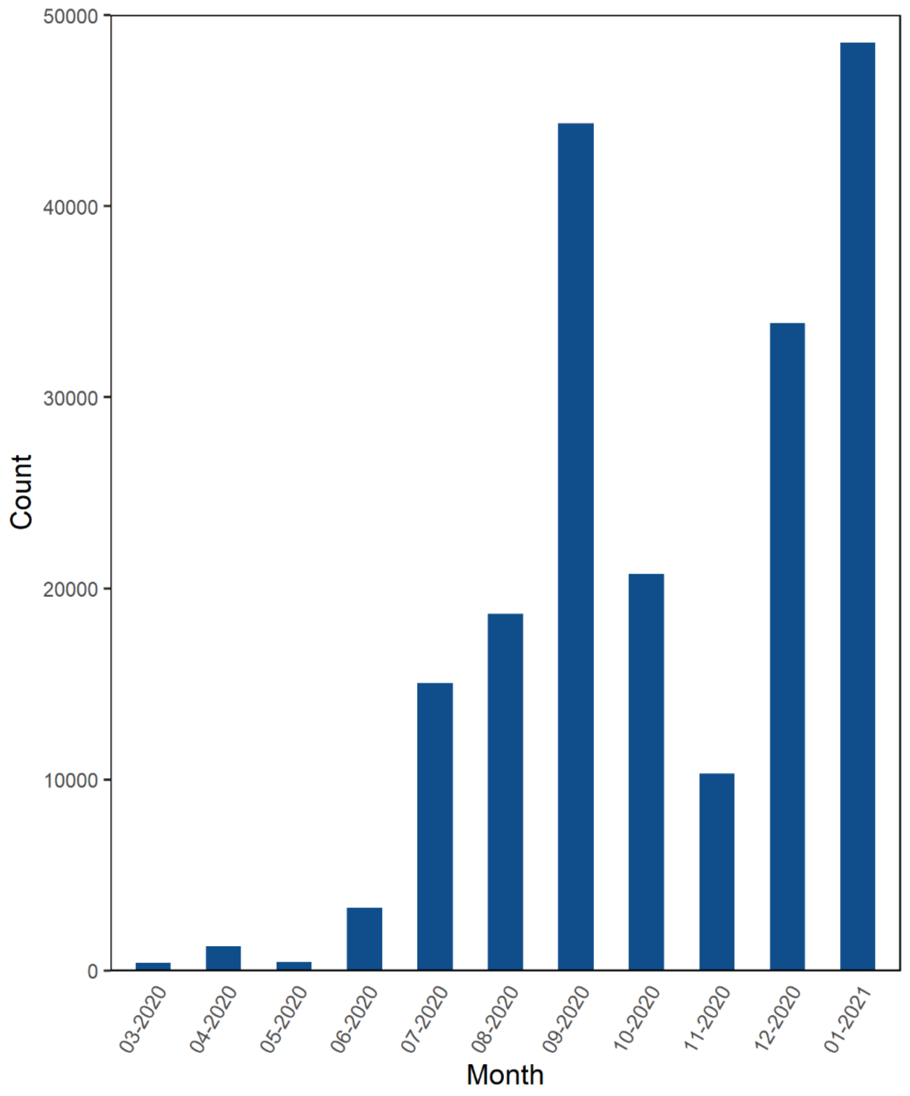

Figure S1. Number of patients with positive PCR in the COVID-19 cohort by month during the study period.

Table S1. ICD-10 codes for cardiac risk factors.

Diagnosis	**ICD-10 code**
Obesity	E66, E66.01, E66.2, E66.3, E66.9, E67
Diabetes Mellitus	E08.42, E08.65, E08.9, E10.10, E10.29, E10.311, E10.40, E10.65, E10.9, E11.00, E11.21, E11.29, E11.311, E11.40, E11.51, E11.618, E11.641, E11.65, E11.8, E11.9, E13, E13.1, E13.3, E13.31, E13.35, E13.36, E13.4, E13.69, E13.8, E13.9
Hyperlipidemia	E78, E78.0, E78.1, E78.2, E78.4, E78.9
CKD	I12.0, N18, N18.2, N18.3, N18.4, N18.5, N18.6, N18.9, N19
PVD	I73, I73.9
Essential hypertension	I10
ACS	I20.0, I21, I21.0, I21.09, I21.1, I21.11, I21.19, I21.2, I21.29, I21.3, I21.4, I23.0, I24.0, I24.8, I25.1
CVA	G45, G45.0, G45.4, G45.9, I63.019, I63.139, I63.30, I63.40, I63.50, I67.89
Heart failure	I09.81, I50, I50.1, I50.2, I50.21, I50.22, I50.23, I50.3, I50.30, I50.32, I50.33, I50.42, I50.43, I50.9

CKD = chronic kidney disease. PVD = peripheral vascular disease. CVA = Cerebrovascular Accident, ACS = acute coronary syndrome. HF = Heart Failure.

Table S2. Crude and adjusted HRs for myocarditis.

	Univariable model		Multivariable model	
	HR (95% CI)	P Value	aHR (95% CI)	P Value
COVID-19	1.00 (0.47-2.13)	1.000	1.08 (0.45-2.56)	0.869
Age	0.98 (0.96-1.00)	0.124	**0.96 (0.93-1.00)**	**0.045 ***
Sex (male)	**4.09 (1.86-8.97)**	**<0.001 ***	**4.42 (1.64-11.96) ***	**0.003 ***
BMI	1.01 (0.99-1.02)	0.595	1.00 (0.97-1.04)	0.935
Diabetes	1.21 (0.47-3.12)	0.689	1.15 (0.26-5.00)	0.856
Hyperlipidemia	0.60 (0.27-1.32)	0.205	0.33 (0.08-1.43)	0.139
Obesity	**1.99 (1.03-3.87)**	**0.041 ***	2.31 (0.99-5.41)	0.053
CKD	**3.53 (1.25-9.99)**	**0.017 ***	3.80 (0.82-17.66)	0.088
Smoking (Now)	1.59 (0.67-3.78)	0.295	1.62 (0.65-4.06)	0.304
Smoking (Past)	1.55 (0.58-4.14)	0.387	1.85 (0.61-5.64)	0.277
PVD	4.07 (0.98-16.94)	0.054	1.35 (0.14-12.84)	0.793
ACS	2.53 (0.99-6.52)	0.054	3.93 (0.76-20.40)	0.104
Hypertension	1.07 (0.47-2.45)	0.870	1.46 (0.36-5.87)	0.592

CKD= chronic kidney disease. PVD= peripheral vascular disease. ACS= acute coronary syndrome. * Statistically significant results are highlighted in bold.

Table S3. Crude and adjusted HRs for pericarditis.

	Univariable model		Multivariable model	
	HR (95% CI)	P Value	aHR (95% CI)	P Value
COVID-19	0.63 (0.33-1.22)	0.171	0.53 (0.25-1.13)	0.100
Age	**1.02 (1.00-1.03)**	**0.019 ***	1.01 (0.99-1.03)	0.537
Sex (male)	**1.89 (1.14-3.14)**	**0.014 ***	**1.93 (1.09-3.41)**	**0.025 ***
BMI	1.00 (0.99-1.02)	0.625	1.00 (0.94-1.06)	0.897
Diabetes	**1.95 (1.06-3.59)**	**0.032 ***	0.97 (0.43-2.21)	0.950
Hyperlipidemia	**1.79 (1.09-2.94)**	**0.021 ***	1.13 (0.54-2.38)	0.749
Obesity	1.40 (0.83-2.36)	0.213	1.32 (0.64-2.71)	0.448
CKD	**4.09 (1.95-8.59)**	**<0.001 ***	1.91 (0.72-5.05)	0.191
Smoking (Now)	1.13 (0.56-2.27)	0.727	0.85 (0.42-1.74)	0.661
Smoking (Past)	1.23 (0.57-2.63)	0.597	0.73 (0.33-1.63)	0.439
PVD	**7.23 (3.12-16.78)**	**<0.001 ***	**4.20 (1.50-11.72)**	**0.006 ***
ACS	**3.31 (1.73-6.34)**	**<0.001 ***	1.52 (0.61-3.80)	0.366
Hypertension	**1.77 (1.02-3.06)**	**0.041 ***	0.88 (0.38-2.06)	0.770

CKD= chronic kidney disease. PVD= peripheral vascular disease. ACS= acute coronary syndrome. * Statistically significant results are highlighted in bold.
